# 

**Published:** 1897-07-24

**Authors:** 


### EARLY EDITION.

**Notices of Births, Deaths, and Marriages** are inserted in this column at a charge of 2s. 6d. for an announcement not exceeding four lines.

### NOTICE TO CORRESPONDENTS.

All MS., Letters, Books for Review, and other matters intended for the Editor should be addressed The Editor, "The Hospital," The Hospital Building, 28 & 29, Southampton Street, Strand, W.C.

The Editor cannot undertake to return rejected MS., even when accompanied by stamped directed envelope.

**Notice to Advertisers and Subscribers.**

All Advertisements, Orders for Copies of The Hospital, and Business Communications should be addressed to **THE MANAGER, (Not to The Editor),** The Hospital, The Hospital Building, 28 & 29, Southampton Street, Strand, London, W.C.

The Cost of Subscription, post free, is as follows:—Per week, 2½d.; per quarter, 2s. 8d.; per half-year, 5s. 3d.; per annum, 10s. 6d. Foreign:—Per annum, 15s. 2d.; payable, in all cases, in advance.

### BOOKS RECEIVED.

THE F. A. DAVIS CO.

"Eye Strain in Health and Disease." By Ambrose L. Ranney, A.M., M.D.

H. K. LEWIS.

"Reports of the Medical, Surgical, and Pathological Registers of the Middlesex Hospital."

"DEVIZES GAZETTE" OFFICE.

"Annual Report of the Wilts County Asylum."

J. R. WILLIAMS AND CO., LIVERPOOL.

"Report on the Health of Liverpool during 1896." By E. W. Hope M.D.

**Periodicals and Pamphlets Received.**—Charity Organisation Review, New Age, Women's Signal, Dietetic and Hygienic Gazette.

### SCRAPS AND GLEANINGS.

Dundee hold a Hospital Saturday collection this year.

Payments by in-patients at the Boscombe Hospital have now been abolished.

The Demilt Dispensary, New York, treated 31,494 new and 44,667 old patients in 1896.

Fifty-nine in, and 1,203 out-patients were treated at the Halifax Eye and Ear Hospital in 1896.

There were 398 subscribers to the Surbiton Cottage Hospital last year, their contributions amounting to £453.

It is stated that there are 20,000 deaf and dumb persons in the United Kingdom, 2,000 of whom are resident in London.

Donegal County Infirmary, at Lifford, treated 302 in-patients in the year 1896-7, of whom 197 were males and 105 females.

Annual subscriptions to the Hambrook Village Hospital have fallen off during the last 10 years. In 1887 they amounted to £82; in 1896 to £65.

Although the patients treated at the Stockport Infirmary (4,941) have increased in 1896 by nearly 100, the expenditure has been decreased by about £128.

During the eleven months ended April 30th, 1897, 122 patients were treated at the Retford Cottage Hospital, and 976 at the Retford Dispensary.

Three hundred and eighty-five patients were admitted to the Suffolk Convalescent Home at Felixstowe during the nine months the home was open in 1896.

In the first year after its establishment (1882) the Children's Fresh Air Mission sent 269 children into the country. Last year (1896) the number reached 3,330.

The three institutions known as the Nottingham and Notts Convalescent Homes admitted 781 patients during 1896; an increase of 29 on the previous year.

The income of the Falkirk Cottage Hospital for the year ended March 31st, 1897, was £604, and the expenditure was £609. 137 patients were treated, as compared with 120 in the previous year.

The St. Luke's Homœopathic Hospital, which was opened in Philadelphia in January, 1896, in the thirteen months ending February, 1897, treated 303 in-patients, and 3,441 dispensary patients.

The income of the Warrington Infirmary for the year 1896 was £2,279, against £2,212 in 1895, and the expenditure was £2,095 against £2,252. 434 in and 4,5[illegible]2 dispensary patients were treated.

The year 1896 has been a busy one at the Grimsby and District Hospital. Not only have extensive alterations and additions to the building been made, but an entirely new system of drainage has been laid down.

During the year 1896 the After Care Association, which aims at facilitating the readmission of poor male and female convalescents from lunatic asylums into social life, assisted 135 cases, as compared with 121 in 1895.

The Paisley Royal Victoria Eye Infirmary had a large increase of work in 1896 when compared with that of the previous year; 103 in-patients, and 3,203 new out-patients were treated, an increase of 35 and 232 respectively.

The average cost per patient per week at the Charnwood Forest Convalescent Home in 1896 was 5s. 1½d. for board and 5s. 8½d. for maintenance, or 10s. 10d. in all. The cost in 1895 was 6s. 3d. and 5s. 2¼d. respectively, or 11s. 5¼d. in all.

While the subscription list at that excellent institution, the Mary Wardell Convalescent Home for Scarlet Fever shows signs of falling off, the amounts received from the patients show an increase from £856 in 1890 to £1,490 in 1896.

Of the 1,727 patients admitted into the Hospital Convalescent Home, at Swanley, 455 came from the London Hospital, 299 from St. Thomas', 297 from Guy's, 266 from the Middlesex, 215 from the Westminster, and 195 from St. Mary's Hospitals.

The daily average number of in-patients treated at the Torres Straits Hospital at Thursday Island has risen from 7·7 in 1894 to 10·9 in 1895, and to 19 in 1896. The total number of patients treated in 1896 was 637, viz., 347 in and 290 out patients.

Seventy-three in-patients were admitted to the Coleraine Cottage Hospital in 1896; 20 were refused owing to want of space; and 15 out-patients were attended by the hospital staff. One hundred and fifty-seven cases were also visited by the district nurse.

During the year 1896 16,941 patients availed themselves of the benefits of the Leicester Infirmary, viz., 2,892 in and 14,049 out patients. The average stay of each in-patient was 24 days, as compared with 25¼ days in 1895. The average cost per day was 2s. 9d., and per out-patient 2s. 1½d.

The poorest women and girls, who are employed chiefly in factories and workshops, are sent by the Factory Girls' Country Holiday Fund into the country or to the seaside for a week or longer during the summer months. In 1896 1,050 women and girls enjoyed a holiday through the assistance of the fund.

The number of cases admitted into the Grahamstown Asylum Cape Colony) in 1896 was 119, of whom 60 were Europeans. Altogether 344 cases were under treatment during the year. At the Grahamstown Chronic Sick Hospital, a part of the same institution, 199 were under care in 1896, of whom 146 were Europeans.

Fifteen patients were admitted to the Oakeley Hospital at Blaenau Festiniog during 1896, of whom five were seriously injured.

The annual report of the Worcester Infirmary states that in 1896 the daily average number of in-patients was 90·8, and of the household 49·2. The cost per bed was £46 0s. 3½d., against £47 11s. 0¾d. in 1895.

The average term of residence of in-patients at the Essex and Colchester Hospital during 1896 was 34 days, which is less by 5¾ days than in 1895, a movement in the right direction, which, we hope, will be followed up in the current year.

The donation and subscription list of the Horsham Cottage Hospital for 1896 shows a falling off of some £60.

About £8,300 has been received towards building and financing the Kettering and District Cottage Hospital, of which about £4,500 has been expended in erecting the hospital.

Towards the cot recently opened at the Gateshead Children's Hospital, as one of the local commemorations of the Diamond Jubilee, about £54[illegible] was collected, so that after paying for the cot and incidental expenses there remains a sum of £520 for investment.

### The Student of Medicine.

### APPOINTMENTS.

C. W. Low, M.B.Durh., M.R.C.S.Eng., L.S.A Lond., appointed Medical Officer of Health to the East Stow Rural District Council. W. T. McComb, L.R.C.P., L.R.C.S.Edin., appointed Medical Officer of Health for the Borough of Beccles. H. V. McKenzie, M.D.Edin.. appointed Ophthalmic Surgeon to the Torbay Hospital and Eye Infirmary, Torquay. C. A. Morton, F.R.C.S.Eng., appointed Joint Professor of Surgery in University College, Bristol. — Pearce, M.R.C.S., L.R.C.P., Assistant House Surgeon, appointed House Physician to the Northern Hospital, Liverpool. S. J. Roberts, M.R.C.S., L.R.C.P., appointed Medical Officer for the Third District of the Aston Union. F. Spreat, M.R.C.S.Eng., L.S.A., appointed Medical Officer for the Friern Barnet District of the Barnet Union. L. E. W. Stephens, M.R.C.S.Eng., L S.A., appointed Medical Officer for the Third District of the Havant Union. J. T. Thomas, L.R.C P.I., L.R.C.S.Edin., appointed Medical Officer of Health for the Barrow-on-Soar, Billesdon, Blaby, Gretton, Hallaton, Hinckley, Lutterworth, Monks Kirby, Oakham. and Uppingham Rural Districts; and Melton Mowbray, Thurmaston, and Wigston Magna Urban Districts. W. Tiplady, L.R.C.P., L.R.C.S.Edin., appointed Medical Officer of Health to the Tow Law Urban District Council. D. Wingate, M.B., C.M.Glasg , appointed Medical Officer for Easington District of the Easington Union. A. Auld, M.B., C.M.Glasg., appointed Medical Officer for the Balne District of the Pontefract Union.

### THE LONDON HOSPITAL MEDICAL COLLEGE.

Distribution of Prizes—Session 1896-97.

Price Scholarship in Science: £120, Mr. H. E. Ridewood. Price Scholarship in Anatomy and Physiology: £60, Mr. E. W. A. Walker. Entrance Science Scholarships: £60 scholarship, Mr. A. B. Lindesey; £35 scholarship, Mr. C. E. Ham. Buxton Scholarships—Arts: £30 scholarship, Mr. T. S. Dudding; £20 scholarship, Mr. T. J. Latham. Epsom Scholarship: For students of Epsom College, Mr. E. F. Fisher. Clinical Medicine: £20 scholarship, Mr. F. F. Ward; honorary certificates, Mr. C. B. Wall and Mr. A. G. Wilson. Clinical Surgery: £20 scholarship, Mr. W. E. Heilborn; honorary certificates, Mr. T. S. Novis and Mr. E. E. Prest. Clinical Obstetrics: £20 scholarship. Mr. R. W. Pearson; honorary certificates, Mr. H. L. Gregory and Mr. G. Hutcheson. Duckworth Nelson Prize; Biennial—£10, Mr. T. S. Novis; honorary certificate, Mr. A. G. Wilson. Andrew Clark Prize: Awarded 1896. Letheby Prizes: Senior £20, Mr. H. D. Pollard; honorary certificates, Mr. O. Eichholz, Mr. H. Balean, and Mr. M. F. Reaney; Junior £10, Mr. R. V. Dolbey; honorary certificates, Mr. C. E. Ham, Mr. T. L. Lewis, and Mr. T. S. Dudding. Sutton Scholarship: £20 Scholarship, Mr. E. Frazer. Anatomy and Physiology: £25 Scholarship, Mr. A. F. Tredgold; Honorary Certificates, Mr. W. R. Dunstan, Mr. J. Sherren, Mr. H. D. Pollard, Mr. H. Balean, and Mr. E. Merry. Anatomy and Biology: £20 Scholarship, Mr. H. E. Ridewood; Honorary Certificate, Mr. C. E. Ham. Dressers' Prizes: £15 Prizes, Mr. M. F. Reaney and Mr. A. N. Symons; £10 Prize, Mr. W. B. Taylor; £7 10s. Prizes, Mr. B. C. Broomhall and Mr. R. H. N. Smith; £5 Prize, Mr. H. Hodgson; Honorary Certificates, Mr. J. H. Sanders and Mr. E. M. Ridge. Practical Anatomy: £6 Prize, Mr. A. B. Soltau; £4 Prize, Mr. A. L. Matthews; Honorary Certificates, Mr, A. S. David, Mr. C.L. Traylen, Mr. D. Ellis, Mr. A. N. Symons, and Mr. R. Gauld.

